# The Methods of Fall Detection: A Literature Review

**DOI:** 10.3390/s23115212

**Published:** 2023-05-30

**Authors:** Nishat Tasnim Newaz, Eisuke Hanada

**Affiliations:** 1 Department of Information Science and Engineering, Saga University, Saga 8408502, Japan; 2 Faculty of Science and Engineering, Saga University, Saga 8408502, Japan; hanada@cc.saga-u.ac.jp

**Keywords:** fall detection systems (FDS), fall detection types, IoT, cloud, machine learning, kinematic signals, biomedical signals, FDS future scope

## Abstract

Fall Detection Systems (FDS) are automated systems designed to detect falls experienced by older adults or individuals. Early or real-time detection of falls may reduce the risk of major problems. This literature review explores the current state of research on FDS and its applications. The review shows various types and strategies of fall detection methods. Each type of fall detection is discussed with its pros and cons. Datasets of fall detection systems are also discussed. Security and privacy issues related to fall detection systems are also considered in the discussion. The review also examines the challenges of fall detection methods. Sensors, algorithms, and validation methods related to fall detection are also talked over. This work found that fall detection research has gradually increased and become popular in the last four decades. The effectiveness and popularity of all strategies are also discussed. The literature review underscores the promising potential of FDS and highlights areas for further research and development.

## 1. Introduction

Falls by an older person are a significant public health issue because they can result in disabling fractures and cause severe psychological problems that diminish a person’s level of independence. Falls can be fatal, particularly for the elderly. According to one study [1], falls are the leading cause of injury-related death for seniors aged 79 or over and the second most prevalent cause of injury-related (unintentional) mortality for adults of all ages. A person’s quality of life (QoL) is influenced by their intellectual ability, which has been documented to be impaired when elderly persons become bedridden after falls. A fall detection system is an aid with the main purpose of generating an alert if a fall has occurred. They show great promises of mitigating some of the detrimental impacts of falls. Fall detectors have a substantial impact on how soon assistance is provided after a fall as well as decreasing the fear of falling. Falling and being afraid of falling are related: being terrified of having fallen may increase the likelihood that a person will suffer a fall [2]. Numerous studies have been done to create strategies and methods for improving the functional abilities of the elderly and ill. Some systems use cameras, sensors, and computer technology. Such systems for older persons can both improve the capacity for independent living by enhancing their sense of security in a supportive environment and reduce the amount of physical labor required for their care by reducing the need for nurses or other support employees [3]. The objective of this paper is to document the currently available systems for fall detection and their outcomes, which we hope will be a basis for future research and development of fall detection systems.

## 2. Methodology

**Figure 1:**
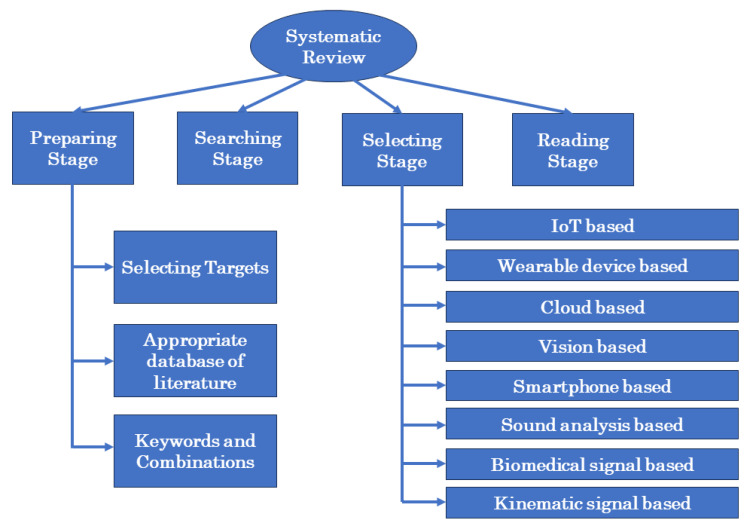
Structure of our systematic review.

Through a search of the databases of PubMed, Google Scholar, and Summon (Ex Libris, Part of Clarivate), we found several publications with articles on falls. To better comprehend the present strategies toward eliminating falls, several kinds of fall were surveyed. We hope this will lead to the creation of algorithms that can be used to develop new systems in the future. Our review of the publications was done in four stages: preparation, searching, selecting, and analysis which is illustrated in Figure 1.

In the preparation stage, we first selected the targets we needed to acquire. In this stage, our main target was to search for all published research about falls. We also searched for appropriate databases and selected Google Scholar, PubMed, and Summon of the Saga University Library. Keywords and combinations were then prepared with which we searched the selected databases. Our keywords for the search were “Fall Detection”, “Fall detection system”, “Fall detection algorithm”, “Fall detection machine learning”, “Fall detection dataset”, “Fall detection video”, and “Fall prevention”. Next, we used the above keywords to search the publications. First, a Google Scholar and PubMed search was done for the years from 1990 to 2022. Over 30,000 articles were shown for “Fall detection/wearable and wireless device/IoT (Internet of Things) based fall detection systems”. From them, we have selected all relevant papers.

- IoT-based.
- Wearable device-based.
- Cloud-based.
- Vision-based.
- Smartphone-based.
- Sound analysis-based.
- Biomedical signal-based.
- Kinematic signal-based.

After these three stages were finished, we began analyzing the 75 selected papers.

## 3. Fall Detection Strategies

**Figure 2:**
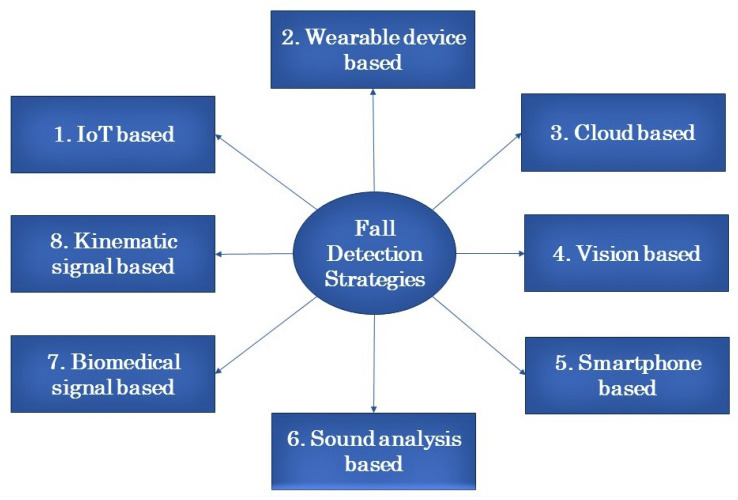
Fall detection strategies.

Sensors and machine learning are vital parts of fall detection and fall recognition. Multiple methods of fall detection used many types of sensors. To acquire the final decision of fall events from the sensor data, machine learning, deep learning, and artificial intelligence are used. Researchers worldwide have shown significant interest in human activity recognition. Over the past few years, numerous approaches have been introduced, focusing on identifying various activities such as walking, running, jumping, jogging, falling, and more. The authors of [4] contributed a valuable resource in the realm of real-time Human Activity Recognition (HAR) by offering a comprehensive reference for real-time fall detection. Fall detection holds particular importance among these activities as it poses a common and dangerous risk for people of all ages, particularly impacting the elderly. Typically, these applications utilize sensors capable of detecting sudden movement changes. These sensors can be integrated into wearable devices such as smartphones, necklaces, or smart wristbands, making them easily wearable. In fall detection strategies, the first step is to recognize human activity, which enables the identification of fall events. Efficient recognition of human activity plays a crucial role in accurately detecting falls. Major strategies of fall detection are illustrated in Figure 2.

### 3.1. IoT-Based

An Internet of Things-based system can also be helpful for detecting falls, and researchers have made significant strides in both the detection and management of falls. Gia et al. [5] introduced a wearable IoT-based system to ensure an appropriate degree of control and reliability for their fall detection system. The wearable nodes are inexpensive, light, and adaptable, but they have limited operational lifespans, suffer from interruptions, and are inconsistent. The system is efficient and feasible, but complicated. Chatterjee et al. [6] proposed an intelligent healthcare system with IoT and a decision support system. The system is low-cost, provides ambient assisted living, and improves the experience of patients. This paper lacks a discussion of its uses in healthcare environments. Tzallas et al. [7] introduced a system named PERFORM that does real-time remote observation, evolution, and management of PD. It provides simple, safe, painless, and non-invasive assistance to the clinician neurologist. The privacy of patient data was not clarified in this paper. Pereira et al. [8] developed a portable mobile application to support individual PD patients. It offers information and support for professionals working with patients and their caregivers, but has a lack of discussion about feedback management. Wankhee et al. [9] discussed the action of a pupil sensor monitor and head motion network attached to an IoT Infrastructure. ALS patients with this system can manipulate equipment and safety control doors. This paper is focused on devices, not on open issues. Punin et al. [10] created a non-intrusive equipment-based remote framework with FOG to gather information from PD patients. It has a small body and is light, ergonomic, and within reach of people with limited resources. The system has a good cost–benefit ratio.

### 3.2. Wearable Device Based

Much research has been done on wearable devices that are connected through wireless technology. Pierleoni et al. [11] proposed a system to detect falls that combines a magnetometer, gyroscope, and accelerometer to overcome the limitations of a single accelerometer, but they did not present the data well and only briefly discussed current issues and challenges. Baga et al. [12] offered a system that can manage neuro-degenerative diseases by minimizing the size of wearable sensors. They presented a modular system architecture that can be adapted to any disease. This paper has a good discussion of how to overcome security issues, but is lacking discussion of detection and quantification. Avvenuti et al. [13] proposed a monitoring system through which sensors collect real-time data and process them before sending alerts to a caregiver. It can collect data from sensors that are both wearable and wireless and uses a small bandwidth to send alerts to caregivers. Wang et al. [14] proposed the device-free fall detection model ‘Wifall’ that works through a wireless network without hardware modification or extra environmental setup, but it is efficient only for a single-person environment. LeMoyne et al. [15] successfully included a smartphone as a stage for remote accelerometer machine learning (ML) to integrate three mature systems: for deep brain stimulation, a smartphone, and ML. It needs further development in terms of miniaturization. Bustamante et al. [16] proposed a personal remote monitoring device with a radio frequency (RF) transceiver that has different layers of software and hardware that can work independently. It is used only for Parkinson’s disease (PD) and Alzheimer’s disease patients. Tisch et al. [17] were able to distinguish Alzheimer’s from PD by nano-material sensors and breath tests. Their system is only for detection, not for the management of the disease. Theodoridis et al. [18] introduced a method to detect falls based on a recurrent neural network. It can identify falls without false positive detection while keeping the total system low in cost. This paper lacks a discussion about camera data.

### 3.3. Cloud-Based

Pan et al. [19] suggested a model “PD Dr” mobile cloud-based mHealth application. It tests hand movement while a person is resting, walking, or turning, but does not provide a long-term continual assessment. Al Hussein et al. [20] proposed a framework that transmits a voice signal to the cloud and processes the signal to detect PD. Two different databases and four ML classifiers are used, and the accuracy is up to 97%. The system has not been applied in real-life settings. Depari et al. [21] proposed an architectural model to connect prototype instruments to the cloud that offers low-cost real-time data and stable, accessible, and compatible message-oriented strategies. It cannot be used for interchanging raw or metadata. Ivan et al. [22] proposed an application that collects image data and 3D scenes for transmission to a cloud, only sending the results to the cloud after they have been finalized, which saves bandwidth and makes the network fluffy. It only collects limited types of information. Akhund et al. [23] proposed a wearable device for Alzheimer’s patients that collects various data and sends it to the cloud. The use of a variety of sensors can give good results and the accuracy is quite acceptable. It can only make notifications and detect diseases, not manage them. In [24], the fall detection tools, indications, algorithms, and various fall types were emphasized as important components of their pre-impact fall detection analysis. The efficiency of their detection method was also investigated and reported in terms of its sensitivity, specificity, and detection/lead time. The researchers in [25] presented a robust approach to ensure the security of cloud- and IoT-based systems.

### 3.4. Vision-Based

Kamarol et al. [26] proposed an appearance-based template to perform facial expression recognition in video streams. It performs several state-of-the-art expression-based feature selection methods. Classification is easy, but not checked during complex movements such as dancing, jumping, or other such movements that are not regularly done. Xie et al. [27] provided a tool that uses the histogram sequence of local speech dialects or binary patterns from three orthogonal planes (LGBP-TOPs). The machine does well in detecting simple falls, but the accuracy of the system needs to be improved. Suja et al. [28] proposed a geometrically focused method for the identification of six specific emotions in video sequences that improved accuracy compared to other methods. Evaluation cannot be determined in real-time conditions. Chiranjeevi et al. [29] proposed a method of lightweight vs. emotional classification that serves as a preprocessor of conventional emotion classification approaches. It has the computational benefit of using the suggested approaches as a preprocessing device. It also works well under sudden postural variations. Shojaei et al. [30] proposed a method called severe sparse learning, capable of jointly studying a dictionary and a non-linear model of classification. Under challenging scenarios, it gives a good performance. The computational cost is high for both feature extraction and classification. Three ultra-wideband (UWB) radars and a CNN-LSTM deep neural network model with a 90% accuracy were proposed by [31] to detect falls in a real, 40 square-meter apartment. They supported the notion that UWB radars are a rapidly developing technology that enables the detection of falls as well as the identification of activities. The researchers of [32] used surveillance camera footage in a real-time approach that is efficient and precise at identifying persons who are falling. Eight current methods were compared to the proposed system’s performance on the publicly accessible fall detection datasets Multiple Cameras Fall and UR Fall Detection, which when used in experiments have generated respective accuracy results of 99.2 and 99.25. Other publicly accessible standard datasets were also tested. Marcos et al. [33] presented a method to improve a system’s independence, reduce the hand-engineered image processing processes, and make the system flexible. Optical flow images are used as the network’s input to represent the motion of the video and make the system scenario independent. Datasets are required to train the system. This system cannot work with multiple people. Zerrouki et al. [34] proposed an approach to detect falls that depends on variations in the pattern of the silhouettes of humans. It identifies human postures by curvelet transform and reduction of the feature vector and uses SVM (Support Vector Machine) to classify falls and non-falls. Unfortunately, it does not work properly in the dark. Liu et al. [35] proposed a fall detection system based on KNN (K Nearest Neighbor). The system utilizes off-the-shelf devices for detection and classifies posture using a human body silhouette. The main advantage of this system is that privacy is protected: only the dimensions of the human body’s silhouette are maintained. This system is only useful for upper limb activities and situations in which there is a change of posture. Sase et al. [36] introduced a method for detecting falls using depth videos. Their method identifies falls by comparing the estimated ROI to predefined thresholds. Its weakness is that it is not applicable if the person is lying down. O. Keskes et al. [37] suggested a system based on a broad vision that uses the ST-GCN algorithm to prove the action recognition field. The performance of the algorithm shows reliable, efficient, and robust results for detecting falls. This system uses data sets to solve problems related to insufficient data. Rougier et al. [38] presented a technique for detecting falls in older people that focuses on the diversity of the human shape and stores their movements. It offers encouraging outcomes on video clips of everyday activities and realistic accidents. It is a motion-based system, so the video-based method infringes on privacy. It also uses artificial vision techniques. Miguel et al. [39] proposed a low-cost fall detector for smart homes. This detector uses several different algorithms as input to a machine learning algorithm, which produces highly accurate detection results. It is a low-cost device that is not worn and only works in daylight. Foroughi et al. [40] identify numerous conventional geriatric monitoring applications and use posture-based events in a home surveillance situation. Their consistent experimental success indicates great potential for recognizing various types of falls, including sideways, reverse, and front falls. The range is only 4–5 m. Y.M. Galvão et al. [41] suggested a reliable fall detection system that performs well while interacting with fresh data. Using skeletal information, the Kinect v2 camera can detect up to two people in the same frame. It uses data on the subject’s natural form and three open datasets that concurrently capture geographical and symbolic data. The datasets used lack large-scale training samples.

### 3.5. Smartphone Based

Smartphones currently play a vital role in our daily life. They can be helpful for detecting falls and notifying others about a fall. Mostarac et al. [42] created a system that can detect falls using three-axis accelerometric data. It has low power consumption, good portability, and is of small size. This paper has a lack of discussion about the post-fall condition. Fang et al. [43] displayed a fall detection prototype that has been implemented on an Android-based platform. The suggested system consists of three parts: sensing the accelerometer data from the mobile embedded sensors, understanding the link between fall behavior and the acquired data, and sending a message to predefined contacts when a fall is detected. The findings demonstrate a 72.22% sensitivity and a 73.78% specificity for identifying falls originating from human activities, including sitting, walking, and standing. It is a low-cost fall detection system that works with readily purchased products and wireless technologies, eliminating the need to modify gear, set up the surroundings, or wear extra sensors. It also shows the location of the devices. In [44] by Stone, E.E., researchers developed a fall detection system for older people living at home that utilized Microsoft Kinect and a two-stage detection system in which the first stage analyzes a person’s vertical status in each depth image frame. There is also a vertical status time series that is produced by following the person over time, with on-ground follow-up. The second stage computes confidence that a fall preceded an on-ground occurrence using an ensemble of decision trees. A certain degree of characterization of system performance under real-world circumstances is possible because of the substantial data collected. Cross-validation findings are presented for standing, sitting, and lying down postures, near (within 4 m) vs. far fall locations, and true falls vs. non-falls. Using inexpensive technology, real-time operation is feasible, but the sensor must be able to detect the fall. [45] employed mobile phones as a platform for constructing a fall detection system termed PerFallD. They constructed several detection algorithms for situations both with and without basic peripherals based on mobile phone platforms. They made a comparison of PerFallD’s performance with that of other current research results and a commercial product, with PerFallD delivering good detection performance and power efficiency, according to the testing results. Vallabh et al. [46] classified the difference between the causes of falls and daily activities. Naive Bayes, k-NN, ANN, SVM, and LSM are used to classify the method and show accuracy and precision. This paper is not about fall detection and does not give suggestions for promoting higher accuracy of detection or for any kind of fall prevention.

### 3.6. Sound Analysis-Based

Al Mamun et al. [47] proposed a cloud-based framework for patients from rural areas to obtain instructions from doctors by sending voice recordings via cloud-based systems. Doukas et al. [48] proposed an architecture to detect falls via fall sounds and patient motion. Fall detection is possible from the voice of a person who falls, and notification can be given in times of emergency. The system uses sensors affixed to the body, which is the main demerit of this work. M. Pham et al. [49] proposed a smart home plan to monitor a patient’s activity and give a proper report to a caregiver. The perfect home setup helps to collect appropriate data for processing. It will not provide any help in the event of an emergency. E. Principi et al. [50] suggested architecture for integrating audio signals at the time of an emergency. The system sends abnormal movements of the person to their caregivers. There is also a home automation system for emergencies that operates by giving voice commands. Successful detection of falls by voice and/or sound analysis will be a valuable topic for research. The research of [51] presents a unique, non-wearable, non-intrusive, and adaptable fall detection approach based on an autonomous mobile robot that carries a built-in microphone. The suggested solution takes advantage of environmental audio captured in people’s houses, particularly in their bathrooms. It creates a solution based on a Transformer architecture that receives loud sound input from bathrooms and classifies it with an accuracy of 0.8673 into fall/no-fall classes. In the study of [52], the researchers suggested a new combination of integrated features that includes melcepstral coefficients, gammatone cepstral coefficients, and spectral skewness. To evaluate the classification performance for both binary and multi-class problems, they utilized a decision tree. The method achieved an accuracy rate of 91.39%, precision rate of 96.19%, recall rate of 91.81%, and F1-score of 93.95%. They also compared the method with existing state-of-the-art methods. The experimental results indicate that the method is a reliable choice for fall detection in various settings, including medical centers, nursing homes, senior housing, and healthcare facilities.

### 3.7. Biomedical-Based

In [53], researchers propose a model-based fall discrimination method that uses a microwave Doppler sensor. They modeled human falls mathematically in their simulation and compared the measurement waveform and the observed waveform to detect falls, with high accuracy. Thome et al. [54] proposed an application for automatic fall detection and future pervasive health monitoring. LHMM (Layered Hidden Markov Model) helps to solve the problem of inference. It is robust to low-level step errors and independently extracts features. One problem is that there is no collaboration between points of view. Hwang et al. [55] developed an approach for detecting falls that utilizes signals acquired from a system linked to the chest. It is specifically designed for long-term and ongoing ambulatory surveillance of seniors in crisis situations and real-time monitoring that can distinguish falls from daily activity. Its use is limited because Bluetooth has a small range. The study [56] proposed an algorithm that utilizes pre-trained convolutional neural networks, specifically AlexNet and GoogLeNet, to differentiate between fall and no-fall scenarios using electrocardiogram (ECG) signals. The ECGs for both falling and non-falling cases were collected from eight volunteers. To test the proposed algorithm’s robustness, the researchers augmented their experimental dataset by incorporating two publicly available datasets. The second model developed through transfer learning achieved a high accuracy of 98.44% in classifying falls, daily activities, and no activities. This model was trained using real images and applied to medical images. The researchers of [57] designed a smart cane with remote ECG and fall detection capabilities. The cane includes a self-developed ECG detection circuit, a fall detection module with a gyroscope and accelerometer, and wireless transmission modules. The hardware is divided into two parts: ECG detection using a copper column-shaped detector and fall detection using a gyroscope and accelerometer. The system can be used in real-time monitoring and provide reference data for healthcare professionals and nursing personnel.

### 3.8. Kinematic-Signal-Based

Hidden Markov Models (HMMs) have been successfully applied in fall detection systems due to their ability to model temporal dependencies in sensor data and to handle noisy or incomplete sensor readings. HMM-based fall detection systems have shown promising results in accurately detecting falls and reducing false alarms. Ref. [58] is an informative and well-written paper that provides a comprehensive overview of the Hidden Markov Model (HMM) and its applications in human activity recognition and fall detection. The authors have discussed the strengths and weaknesses of the HMM approach and highlighted several key studies that demonstrate its effectiveness. Overall, this paper is an essential resource for researchers interested in the field of activity recognition and fall detection. Ref. [59] is a well-written and insightful paper that provides a comprehensive review of the latest techniques in biosignal processing and activity modeling for multimodal human activity recognition. The authors have discussed the challenges and opportunities in this field and highlighted several key studies that demonstrate the effectiveness of these techniques. This paper is a valuable resource for researchers interested in developing advanced multimodal activity recognition systems. The researchers in [60] presented a recent method for detecting and recognizing falls using Hidden Markov Models (HMMs). This technique is straightforward, easy to understand, and can be applied to various scenarios, resulting in significant enhancements in machine learning (ML) efficiency. In the study of [61], a wearable device with a single triaxial accelerometer was used to construct a fall detection system. The system tracks human movement using a powerful quaternion algorithm to detect falls from routine everyday activities, then automatically transmits a request for assistance to the caretakers along with the patient’s location. The system’s extremely effective algorithm and low power-consumption hardware design may help the durability of this device. The wearable hardware and software designs are appropriate for outdoor applications. In the novel design in [62], which uses a Kinect sensor to generate a fall alarm, an accelerometer is employed to signal a potential fall. It is relatively inexpensive, but also dependable. They identify characteristics simultaneously from the depth maps and point clouds to extract discriminative fall descriptors. They also demonstrated how a depth sensor can accurately differentiate between these filtered events and falls. Ref. [63] presented the creation of a threshold-based algorithm that uses a bi-axial gyroscope sensor to automatically distinguish between falls and normal activities of Daily Life (ADL). The gyroscope signals were obtained from the ADLs of older people at home and from simulated falls carried out by healthy young volunteers. Young respondents simulated falls, whereas senior subjects were asked to do their usual ADL. These tests were carried out with people wearing a bi-axial gyroscope sensor, with an identical sensor set-up used in each. The system in [64] used two tri-axial accelerometers at different body positions to distinguish between standing, bending, sitting, lying, and four different types of static postures. Dynamic transitions are movements made between these still positions. It is possible to quantify linear acceleration and angular velocity to evaluate whether motion changes are deliberate. A fall event is diagnosed if the transition leading up to a prone or supine position is not deliberate. They provide a fall detection algorithm that can lessen both the false positives and false negatives (for instance, if a person sits down quickly). The research uses triaxial accelerometric data at the waist, wrist, and head to calculate acceleration thresholds for fall detection. There were only two test volunteers in this pilot research, so the results are considered preliminary. They plan to do more experimental investigations that include more subjects, ideally with middle-aged or older volunteers to provide conclusive evidence. The researchers of [65] concluded that, when employing basic thresholds and posture detection, the head and waist are relevant locations for accelerometric fall detection. In contrast, the wrist was not viable for detecting falls. Based on the symmetry principle, Chen et al. [66] proposed a method for classifying falls. Three crucial factors were used for the classification: the rates of various types of falls, action at the major joints, the angle between the body’s midline and the ground, and the proportion of the human body’s breadth to height. It identified both actions related to falls and actions involved in standing up after a fall. Such video-based methods are weak in protecting privacy.

## 4. Datasets of Fall Detection

Researchers have created various types of datasets for the detection and analysis of falls. Among them, the tFall, UMAFall, UPFall, MobiFall, and DLR datasets are the most prominent. The researchers of [67] developed the UP-Fall Detection dataset, a public multimodal dataset for fall detection. Studies on modality techniques for fall detection and classification are needed. They also offered a fall detection system that relies on a 2D CNN inference approach and numerous cameras to examine pictures in defined time frames as well as retrieving attributes utilizing an optical flow method that gathers details on the overall velocity in two successive pictures. When compared to state-of-the-art approaches using a simple CNN network architecture, the suggested multi-vision-based methodology identifies human falls with an accuracy of 95.64%. The researchers [68] studied acceleration-based fall detection utilizing peripheral accelerometers or cell phones. Many studies utilize this unique dataset, which has gathered data on various situations. The system utilizes two distinct classification algorithms and evaluates the datasets as either raw values or with modified values so that the baselines have equivalent circumstances. The researchers use the tFall, MobiFall, and DLR datasets and test them with multiple ML algorithms to see which one gives better accuracy. Among them, the tFall dataset gave the best accuracy. In [69], researchers said that UMAFall is a novel dataset of movement traces obtained by the methodical simulation of a set of preset ADLs, including falls. The UMAFall dataset includes five wearable sensors placed at five separate points on the subjects’ bodies to record motion. It identifies three types of falls by tracing the movement of 17 experimental objects. The tracks contain acceleration and gyroscopes and include magnetometer data collected concurrently from four Bluetooth-enabled sensor motes as well as signals sampled by an accelerometer incorporated in a smartphone, which serves as the data sink for a wearable wireless sensor network. The study of [70] introduces a dataset of human activity that can be used to evaluate novel concepts and make impartial comparisons between various algorithms for fall detection and activity recognition based on smartphone inertial sensor data. The dataset includes signals captured from a smartphone’s accelerometer and gyroscope sensors for four kinds of falls and nine different everyday activities. The problem with this dataset is that the “inactivity” time could not be validated; therefore, there is only a brief amount of data left after a certain activity. Based on variations in the geometry of human silhouettes in vision monitoring, the research of [34] suggested a novel method for accurately identifying fall events. The curvelet transformation and area ratios are used to accomplish this task of identifying human posture in photographs. Additionally, the differential evolution method is used to minimize the feature vector dimension while recognizing postures with a support vector machine. Experimental findings have been reported on several “Fall Detection” datasets. The UP-Fall Detection Dataset is presented in [71]. It consists of raw data and movement feature sets of 17 young healthy people who had three trials of each of 11 activities and falls. Additionally, the collection compiles more than 850 GB of data from vision systems and wearable and environmental sensors. Two test use scenarios were presented. The goal of the dataset is to assist machine learning and human activity identification research groups in properly comparing various fall detection techniques. Additionally, it offers a variety of experimental opportunities for machine learning and video, including pattern identification and communities. The researcher of [72] proposed an impressive research paper that introduces a comprehensive dataset for human activity recognition using acceleration data from smartphones. The authors have presented detailed statistics, experimental setups, and benchmark results that showcase the effectiveness of the proposed dataset. Overall, this paper is an essential contribution to the field of human activity recognition and provides a useful resource for future researchers to evaluate their models.

## 5. Discussions

### 5.1. Cloud Security of Fall Detection Approaches

Khan et al. [73] proposed a secure architecture with wireless body area networks (WBANs) where sensors communicate with bio-metric keys. Data communication maintains privacy and security by generating multiple bio-metric keys that are quite expensive to use. Chen et al. [74] proposed an encrypted cloud-based system with a wearable device that is flexible. User data are separated in such a way that there is adequate security and malicious attacks can be prevented. It is a one-way process that cannot give instructions to the patient. Hamid et al. [75] reported on the safe storage of private health details in the cloud that uses a fog storage facility. The encryption process can create a symmetric key. A weakness is that communication with others cannot be done in times of emergency. Kliem et al. [76] reported a framework for appropriate security for client infrastructure. It provides security for both the client and the network. The system has problems when applied to daily life.

### 5.2. Fall Detection for Older Persons

Every year, one-third of people over 65 experience falls, which has a range of negative effects on their physical and mental health as well as a financial impact on the government [77]. To date, several types of research on a variety of sensors and algorithms have been carried out with the goal of resolving this issue, each with certain constraints. The non-intrusive fall detection system for controlled settings that are presented in [78] is based on a thermal sensor array and is designed for elderly persons living alone. Using very low-resolution thermal sensors, it evaluates the effectiveness of three types of recurrent (RNN) systems for the detection of falls in controlled surroundings. The implemented algorithms are long short-term memory (LSTM), gated recurrent units (GRUs), and Bi-LSTM, with the last one producing the best results, with 93% accuracy. The installation of this system in managed homes for older people could be a significant contribution to their care, allowing timely assistance and lower costs in health centers for prolonged hospitalizations. However, some drawbacks of this work include its susceptibility to uncertainty in terms of ambient temperature and the presence of objects in the coverage area. The authors of [79] showed an early fall detection approach for elderly people. The study in [80] describes a fall detection device that keeps an elderly person under constant watch. The system has two key parts. For use within a 100-foot radius, there is a wearable device with mobile phone communication capabilities. When a fall is detected, the device alerts the mobile phone, which subsequently alerts the user’s designated contact with details of the fall. The major goal is to eliminate the constant necessity of carrying a phone. The system has a 92% accuracy rate for detecting backward falls and an 83% accuracy rate for detecting sideways falls. Using a single depth camera that tracks the major joints of the human body, the researchers [81] developed a reliable fall detection method. A major advantage is that the system can operate in a room that is completely dark using a depth camera that has an infrared sensor. The motion of the human body and its interaction with its surroundings can be measured. For the key joint extraction, a pose-invariant Randomized Decision Tree (RDT) technique is used that has a low computing cost for training and testing. The 3D trajectory of the head serves as the input for the (SVM) classifier, which determines if a fall motion took place. The signal processing techniques are essential for the efficient application of radar technology for detecting geriatric falls. This article uses actual fall data to show how well the fall detection algorithms work and discusses some of the difficulties in developing new fall detection technologies. It has been demonstrated that time-frequency analysis, such as weights, plays a crucial role in classifying human movement and determining fall traits. Using actual data tests, the effectiveness of feature-based fall detection techniques was proven, and issues with fall detection technology development were also covered [48]. They developed a fall detection algorithm specifically for elderly people that has an accelerometer incorporated into a wearable device. Analyzing physical information and the waveform of acceleration of the timing of falling, they created an algorithm that defines the maximum threshold of falling. Firstly, they compared the daily lives of young and elderly people and found that the maximum combined movement is not the same. This was tested with five subjects in the age range of 20 to 70. They measured the maximum combined acceleration of sitting, standing, walking, and other falling postures, the angle of the body movement, and the condition after falling. The algorithm was verified and gives an accuracy of 98%, with high precision [82]. N. Noury et al. [1] reported their research on methods and strategies that include sensors for the automatic, early detection of falls by older persons. The most noteworthy aspect was how difficult it was to evaluate all of the fall detection systems that are now available and the performances of various solutions without using a consistent paradigm. The authors only did a comparison: they did not give any solutions or new methods.

### 5.3. Fall Detection: The Past, Present, and Future

**Figure 3:**
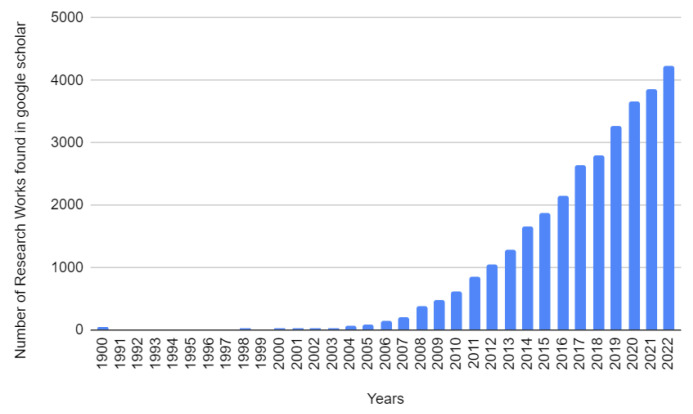
Fall detection research papers found in Google Scholar from 1990 to 2022.

We found numerous research papers in the field of fall detection, with the number of those for each year from 1990 to 2022 shown in Figure 3.

**Figure 4:**
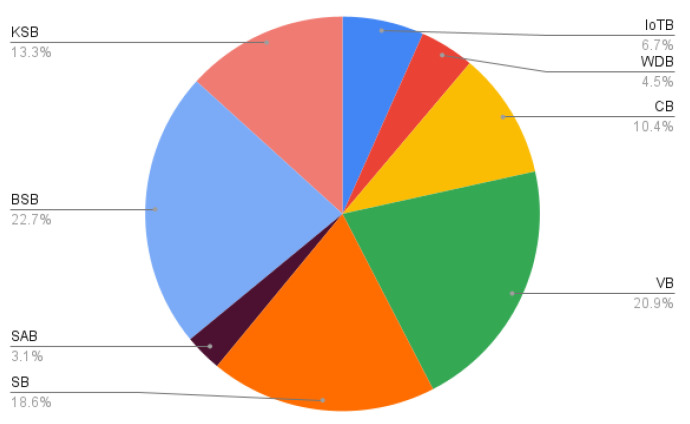
Percentage of the strategies for fall detection among all the research works found from 1990 to the present day.

From this figure, we can see that fall detection is becoming more popular among researchers year by year. These studies report new technologies that can be used to detect falls of elderly people and others. Machine learning and the Internet of Things are widely used. As mentioned in a previous section, voice signal processing can also be used to detect falls, but very few research reports were found related to fall detection that included voice signal analysis. Before 2010, there were very few research papers related to fall detection that studied machine learning and IoT, but since 2016, some excellent papers have focused on machine learning. Though much effort has been made by many researchers in this area, some types of falls are still not detectable, thus more research needs to be done to improve this condition. Machine learning, IoT, and acoustic signal processing are promising areas for fall detection. The percentages of the various strategies used for fall detection found in Google Scholar from 1990 to the present are shown in Figure 4. Here, a total of 1,040,000 related publications were found in Google Scholar.

Explanation of Figure 4:

IoT based (IoTB) = 6.682692308%
Wearable device based (WDB) = 4.480769231%
Cloud based (CB) = 10.38461538%
Vision based (VB) = 20.86538462%
Smartphone based (SB) = 18.55769231%
Sound analysis based (SAB) = 3.067307692%
Biomedical signal based (BSB) = 22.69230769%
Kinematic signal based (KSB) = 13.26923077%

From the chart in Figure 4, we can see that biomedical-signal-based fall detection is the most researched area with 22.69% publications found in Google Scholar among this field. The use of sensors is quite prominent in the detection of falls. The cloud has become more available, and 10.38% of the fall detection systems are now based there. The amount of research based on IoT and deep learning is also quite prominent. Research based on wearable devices is currently becoming less popular because of their inconvenience. Fall detection and recognition with sound analysis is critical and has scope for future research.

## 6. Conclusions

Much research has been done on detecting falls of various types, with various degrees of success. This paper represents an overview of fall detection research done in the last three decades. We identified many methods of fall detection and discussed the results, including which methods of fall detection are the most useful and which require further development. Fall detection systems are gaining more attention from researchers year by year. The knowledge provided by these reports gives us a roadmap of how we can develop more efficient systems that can detect all types of falls, which will benefit mankind greatly in the near future. 

## Data Availability

As it is a review paper, all data we used can be available from public databases. Through a search of the databases of Pub-Med, Google Scholar, and Summon (Ex Libris, Part of Clarivate) we found several publications with articles on falls, and we review those publications and write this manuscript.
